# Identifying vaccine-hesitant subgroups in the Western Pacific using latent class analysis

**DOI:** 10.1038/s41541-025-01067-3

**Published:** 2025-02-12

**Authors:** Yongjin Choi, Kathy Leung, Joseph T. Wu, Heidi J. Larson, Leesa Lin

**Affiliations:** 1 Department of Infectious Disease Epidemiology, London School of Hygiene and Tropical Medicine, London, UK; 2 Laboratory of Data Discovery for Health Limited (D24H), Hong Kong Science Park, Hong Kong SAR, China; 3 WHO Collaborating Centre for Infectious Disease Epidemiology and Control, School of Public Health, LKS Faculty of Medicine, The University of Hong Kong, Hong Kong SAR, China; 4 The University of Hong Kong—Shenzhen Hospital, Shenzhen, China; 5 The Hong Kong Jockey Club Global Health Institute, Hong Kong Special Administrative Region, Hong Kong SAR, China; 6 Centre for the Evaluation of Vaccination, Vaccine & Infectious Disease Institute, University of Antwerp, Antwerp, Belgium; 7 Department of Health Metrics Sciences, University of Washington, Seattle, WA USA

**Keywords:** Viral infection, Epidemiology

## Abstract

Vaccine hesitancy seriously compromised the COVID-19 vaccine roll-out across the Western Pacific with limited evidence-based recommendations for diverse populations across the region. This study investigates the profile of the vaccine-hesitant populations by using fixed-effect latent class analysis and multi-country survey data collected in 12 countries in 2021 and 2022: Cambodia, Viet Nam, Lao PDR, Japan, Republic of Korea, Malaysia, Philippines, Mongolia, Fiji, Solomon Islands, Tonga and Vanuatu. The analysis identified 9 latent classes: *Stay-at-home mothers*, *High-school-educated employees, High-school-educated older adults, High-school-educated young adults, University-educated employees, University-educated older adults, University-educated young adults, Unemployed, Non-compliant employees*. The probabilities of COVID-19 vaccine acceptance and booster uptake were significantly lower in most of these latent classes, compared to *University-educated older adults*, as the reference group. While each country had unique compositions of latent classes among vaccine-hesitant people, there were also some shared risk groups, such as *High-school-educated employees* and *High-school-educated young adults*, across the countries. The study findings demonstrate the benefits of subgroup analysis in unpacking the complex interplay of characteristics within vaccine-hesitant populations, highlighting the need for customised strategies tailored to each country’s unique profile of vaccine hesitancy.

## Introduction

During the COVID-19 pandemic, heightened vaccine scepticism became a serious public health challenge undermining vaccine uptake in the Western Pacific region (WPR) and around the world^1–6^. Vaccine hesitancy is characterised by a state of indecision around vaccination and was already declared among the top global public health challenges by the World Health Organization (WHO) in 2018^7–9^. However, the unprecedented rapid pace of COVID-19 vaccine development further stimulated fear and suspicion towards the vaccines^10^. Widespread vaccine misinformation increased the prevalence of mistrust in public health recommendations^11,12^ and drove many people to rely on unproven treatments for COVID-19^13,14^.

To design effective interventions for tackling vaccine hesitancy, it is essential to carefully identify the target segments in the population and tailor approaches prioritising the ones with the largest share of vaccine hesitant people^15,16^. Tailoring vaccine campaigns and initiatives to specific groups can be more impactful and efficient in tackling the root cause of vaccine hesitancy within each group than one-size-fits-all strategies^17^. Consequently, identifying the country-specific factors associated with vaccine acceptance and uptake has been an important focus in vaccine research, as these help clarify the underlying dynamics of vaccine hesitancy and inform strategies tailored to diverse contexts^18–25^.

Countries in the WPR require customised approaches that account for their unique histories and varying patterns of vaccine hesitancy^26,27^. The example of the 2017 Philippines vaccine scare, following debates around the safety of the Dengvaxia vaccine, demonstrates the impact of historic vaccine hesitancy around one vaccine having an impact on broader vaccine confidence^28^. As another example, Japan has the lowest levels of confidence in Human papillomavirus (HPV) vaccine globally since the Japanese government’s 2013 decision to suspend the proactive recommendation for HPV vaccination due to widespread concerns about its side effects—concerns that were later proven to be unfounded^29^. Sociodemographic profiles of vaccine hesitancy can also vary. In a study conducted in New Zealand, vaccine-hesitant people were more likely to be younger and less educated^30^, while another study focusing on Japanese people reported that vaccine hesitancy was associated with low socioeconomic status, psychological distress and social solidarity, which means the interdependence between community members^29^. Female respondents were more likely to be hesitant to vaccinate in both studies, however. As such, countries have differing profiles of vaccine-hesitant people.

However, empirical evidence that accounts for the cultural and sociodemographic diversity in vaccine hesitancy in the WPR has been scarce^29,31,32^, despite the reported challenges in vaccine roll-outs across the region^5^. There were several global surveys that examined varying characteristics associated with public vaccine hesitancy, but none of them specifically focused on the WPR^32–36^. Only one article conducted a latent class analysis (LCA) to identify factors associated with booster vaccine uptake among nurses during the COVID-19 pandemic, but it only focused on Hong Kong^37^.

This study aims to identify population segments and compare their COVID-19 vaccine acceptance and uptake in 12 countries in the WPR–Cambodia, Viet Nam, Lao PDR, Japan, Republic of Korea (ROK), Malaysia, the Philippines, Mongolia, Fiji, Solomon Islands, Tonga and Vanuatu–by using a fixed-effect LCA and linear regression. The main purpose of this multi-country study is to help map the varying profiles of vaccine hesitancy across countries in the WPR, particularly including those that have been under studied in the literature. LCA enables a more sophisticated interpretation of data by dividing the study sample into subgroups within which individuals share highly correlated categorical variables. This can help us investigate the unobserved heterogeneity in vaccine hesitancy within typical risk factors (e.g., COVID-19 risk perception and non-compliance with health recommendations) and sociodemographic categories (e.g., age, education and employment status)^38^. For example, people who underestimate the risk of COVID-19 are less likely to accept COVID-19 vaccines^18^, but their willingness to vaccinate may also vary by the extent that they take the known or suspected side effects of the vaccines seriously. Therefore, using LCA, we attempt to narrowly identify vaccine-hesitant subgroups within the population and suggest approaches to customise strategies for promoting vaccination for each country in the post-pandemic era.

## Methods

### Study design and participants

This study used 21,052 responses collected in 12 countries in the WPR. The survey was fielded to investigate the status of vaccine confidence and uptake among adults aged 18 or above across the WPR included two rounds of data collection: between June 11th and August 25th in 2021 (round one) and between May 1st and December 22nd in 2022 (round two). The participants completed the survey either online or by computer-assisted telephone interviews (Supplementary Table 1). They were provided with written or verbal informed consent at the beginning of the survey and not offered monetary incentives. To achieve a representative sample, the sampling was stratified by gender, age and sub-national region. However, the data collection tended to oversample the population with tertiary education when compared to the World Development Indicators provided by the World Bank^39^. Therefore, we applied post-stratification weights to account for this discrepancy in educational attainment by using this benchmark data when producing the descriptive statistics and the LCA results. This project was approved by the Observational / Interventions Research Ethics Committee of the London School of Hygiene and Tropical Medicine (reference number: 26636).

The initial dataset included 1238 respondents (5.88%) with missing values, including ‘do not know/refused,’ in the variables used (see Supplementary Table 2 for more detail). We conducted multiple imputation to account for these missing values by considering that they were more than 5% of the sample and largely concentrated in several countries, including Japan, ROK, Malaysia and the Philippines^40^. The imputation procedure was conducted for each round of survey separately, with 30 iterations. The mice package (Version 3.16.0) in R 4.4.0 was used to conduct this procedure.

### Outcomes and covariates

We examined two binary outcome variables: willingness to accept a COVID-19 vaccine for round one and booster vaccine uptake for round two. Booster uptake was examined using the second round survey by considering that more than 42% of the sample had received the second dose in the data collection period. The original acceptance variable was measured using a five-point Likert scale. The question was: ‘*As the new Coronavirus (COVID-19) vaccines become available, would you accept the vaccine?—For yourself?*’ This measure was dichotomised into one, if respondents answered ‘definitely yes,’ and zero if they answered ‘unsure but leaning towards yes,’ ‘don’t know/prefer not to say,’ ‘unsure but leaning towards no,’ or ‘definitely no.’ This cut-off point was determined due to the majority of responses being ‘definitely yes’ (higher than 60%). We also provided a sensitivity analysis result by coding ‘definitely yes’ and ‘yes’ as one for robustness check. The booster uptake variable was coded as one if respondents had received three or more vaccine doses. Otherwise, they were coded as zero.

We used nine categorical variables, which were known to be associated with vaccine hesitancy and measured in both survey rounds, to identify latent classes: trust in local health care providers (HCPs), COVID-19 risk perception, compliance with three types of health-protective behaviours, age, gender, educational attainment and employment status^24,29,30,41^. Trust in HCPs and the COVID-19 risk perception were measured in four point Likert-scale questions (i.e., strongly agree, agree, disagree and strongly disagree) and dichotomised into one if respondents agreed or strongly agreed and zero if otherwise. Trust in HCPs was measured using the following question: ‘*How much do you trust the local health care providers who would give you a COVID-19 vaccine? Would you say you trust them?*’ The question for the risk perception was: ‘*How strongly do you agree or disagree that the threat from Coronavirus is exaggerated?*’ The health-protective behaviour variables included mask-wearing, washing hands and having guests in house. These were recoded as one if respondents answered that they were doing each behaviour a lot or a little more regularly compared to before the COVID-19 pandemic and zero if otherwise (i.e., about the same, less regularly, or not at all). Ages were categorised into five groups to make sure all groups have more than 15% of the sample. Education was categorised into three groups: less than tertiary, tertiary and Master’s degree or higher. The employment status variable included five categories: employees, students, retirees, stay-at-home parents and others including the unemployed.

### Statistical analysis

We conducted a fixed-effect LCA to identify latent classes of survey respondents by including the nine response variables with country categories and time dummies–i.e., survey rounds–and regression analyses to statistically confirm the differences in the probabilities of vaccine acceptance and uptake among the identified classes^42^. In the LCA procedure, we chose to categorise countries into three groups to manage variability and avoid an excessive number of latent classes while still accounting for the diversity across the countries: South East Asian countries, including Cambodia, Viet Nam, Lao PDR, Malaysia and the Philippines, East Asian countries, including Japan, ROK and Mongolia and Pacific Island countries, including Fiji, Solomon Islands, Tonga and Vanuatu. Assuming that there are variations within each of the observed categories in the response variables used^43^, a typical LCA process iteratively classifies the sample into a given number, calculates the model fit and compares the performance by gradually increasing the number of latent classes to find the optimal number of latent classes^38^. We used the maximum likelihood estimation with the Expectation Maximum (EM) algorithm for estimating the model fit. The final LCA model was selected by considering both the interpretability of the results and the following three metrics: the Bayesian Information Criterion (BIC), as the primary score, the Akaike Information Criterion (AIC) and entropy^44,45^. More specifically, we began with one class model and then increased the number of classes until we found an elbow of points in the fit indices or stopped estimating when identified classes were conceptually not plausible^46^.

As a result, we initially selected the 12-group model, as there was a significant decrease in the BIC and AIC and at the point of 12 classes (see Supplementary Fig. 1). We then combined six of these identified latent classes that shared similar characteristics, such as young adults of ages 18–24 and 25–34, into three classes for better interpretability (see Supplementary Table 3).

There are known limitations of LCA that arise from the probabilistic class assignment, including incorrect naming and potential misclassification bias^47^. A misclassification bias occurs when respondents are incorrectly assigned to a particular category. To mitigate such problems, we first defined classes by combining the original response variables and by focusing on factors that account for 95% or more of the class. We also conducted a robustness check by comparing the main results with the results that exclude misclassified observations (Supplementary Fig. 3).

We then used country fixed-effects linear regression models to statistically examine the differences in the acceptance and booster uptake of COVID-19 vaccines among the identified latent classes. In these models, unobserved country-level variations were fixed and standard errors were clustered at the country level.

LCA procedure was conducted by using R (version 4.23.0) and the multilevLCA package (Version 1.5), while regression analyses were based on STATA 17.0. Statistical differences among the identified latent classes were determined based on two-tailed tests and a 95% confidence level.

## Results

### Latent class analysis

Table 1 presents the characteristics of the study sample. Among 21,052 respondents in the data, 62.46% in the first round answered that they would definitely accept COVID-19 vaccines when they became available, and 55.50% had received three or more doses by the completion of survey in the second round. In the study sample, the vaccination rate was higher than the benchmark data obtained from Our World in Data (Supplementary Table 7)^48^. Most respondents trusted local HCPs (82.76%). Nearly half of the respondents (48.11%) agreed that the risk of COVID-19 had been exaggerated. See Supplementary Table 4 for the unweighted descriptive statistics and Supplementary Tables 5, 6 for the weighted sample characteristics stratified by country.

**Table 1:** Descriptive Statistics (Weighted)

Variables	All respondents (%)	Round 1 (%)	Round 2 (%)	Cramér’s V
Number of observations	21,052	10,314	10,738	
Vaccine acceptance	-	62.46	-	
Not vaccinated	-	-	7.19	
Booster vaccination	-	-	55.50	
Trust local health care providers	82.76	81.99	83.50	0.0234
Those who think that COVID-19 is exaggerated	48.11	47.59	48.60	0.0130
Health-protective behaviours				
Mask wearing	70.44	74.21	66.83	0.0830
Washing hands	79.15	82.17	76.26	0.0755
Having guests	38.96	17.78	59.31	0.4371
Female	50.06	50.30	49.83	0.0003
Age				0.0377
18–24	20.63	20.39	20.87	
25–34	24.82	24.54	25.09	
35–44	20.38	20.23	20.54	
45–54	16.22	15.36	17.04	
55+	17.94	19.49	16.47	
Education				0.0217
Tertiary or lower	79.70	79.23	80.14	
Tertiary	18.13	18.50	17.76	
Master+	2.18	2.26	2.10	
Employment status				0.0353
Employed	57.06	56.74	57.37	
Students	7.68	8.33	7.06	
Retirees	6.02	6.73	5.34	
Stay-at-home parents	15.31	14.66	15.93	
Unemployed or others	13.93	13.54	14.30	

**Fig. 1:**
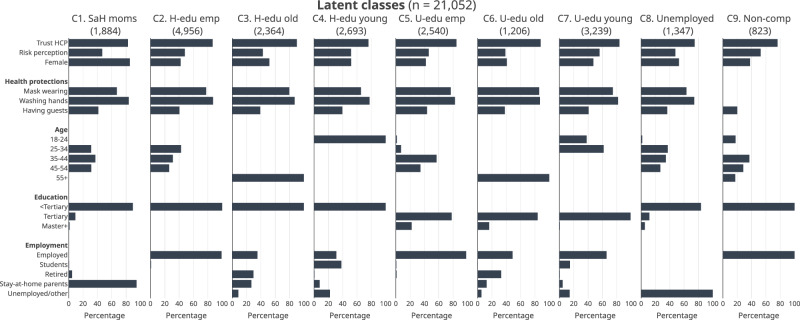
Sample characteristics by latent class. Note. Bars indicate percentage. The figures on top of each plot indicate the numbers of respondents in the latent classes. C1–C9 indicate latent classes: C1–Stay-at-home mothers; C2–High-school-educated employees; C3–High-school-educated older adults; C4–High-school-educated young adults; C5–University-educated employees; C6–University-educated older adults; C7–University-educated young adults; C8–Unemployed; C9–Non-compliant employees.

Figure 1 compares the characteristics of the identified latent classes. Final class memberships for respondents were determined based on their highest probability among the nine classes. The final model yielded nine mutually exclusive classes. *Stay-at-home mothers* are stay-at-home parents who were female. Two were defined by educational attainment and employment status. *High-school-educated employees* were those working full-time or part-time and without completing tertiary-level education, while *University-educated employees* were employees who had completed tertiary- or graduate-level education. Two classes were defined by age among those without tertiary-level education. *High-school-educated young adults* were those who were under 24 years old, while *High-school-educated older adults* were 55 years old or older. Among those with tertiary-level education or higher, *University-educated young adults* were those who were under 34 years old, while *University-educated older adults* were 55 years old or older. *Unemployed* was the class of those who were categorised as unemployed or others, excluding students, retirees and stay-at-home parents. Lastly, *non-compliant employees* were those who were full-time or part-time workers who were not complying with health recommendations against COVID-19.

The size of the latent classes ranged from 823 to 4956 (see Supplementary Fig. 2): (1) *Stay-at-home mothers* (8.95%, 1884 respondents), (2) *High-school-educated employees* (23.54%, 4956 respondents), (3) *High-school-educated older adults* (11.23%, 2364 respondents), (4) *High-school-educated young adults* (12.79%, 2693 respondents), (5) *University-educated employees* (12.07%, 2540 respondents), (6) *University-educated older adults* (5.73%, 1206 respondents), (7) *University-educated young adults* (15.39%, 3239 respondents), (8) *Unemployed* (6.40%, 1347 respondents), (9) *Non-compliant employees* (3.91%, 823 respondents).

### Vaccine acceptance and booster uptake

**Fig. 2:**
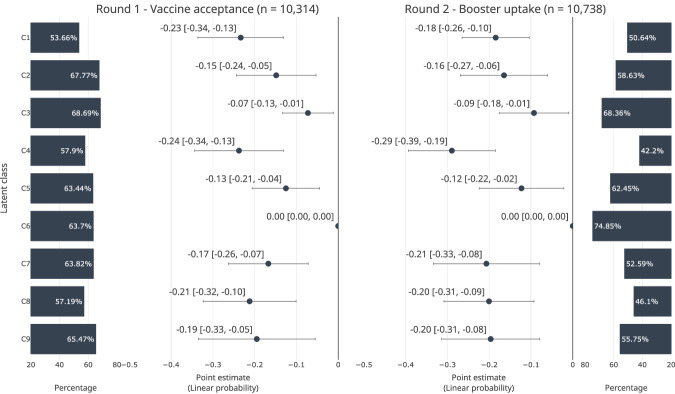
Comparison of COVID-19 vaccine acceptance and booster uptake by latent class. Markers indicate OLS coefficients. Horizontal spikes indicate 95% confidence intervals. C1–C9 indicate latent classes: C1–C9 indicate latent classes: C1–Stay-at-home mothers; C2–High-school-educated employees; C3–High-school-educated older adults; C4–High-school-educated young adults; C5–University-employees; C6–University-educated older adults; C7–University-educated young adults; C8–Unemployed; C9–Non-compliant employees. Horizontal spikes around the markers indicate 95% CIs. Unobserved country-level variations were fixed. Standard errors were clustered at the country level.

Figure 2 reports the perceptages of COVID-19 vaccine accepance in round one and booster uptake in round two (bars) and statisitically examines their differences across latent classes (markers with spikes). Overall, the booster uptake rates in round two generally followed the pattern seen in vaccine acceptance in round one, but with a larger shift among *High-school-educated young adults* (C4), which showed a significant drop in uptake compared to its initial vaccine acceptance. For the first round survey, the percentage of accepting COVID-19 vaccines ranged from 53.66% (C1–*Stay-at-home mothers*) to 68.69% (C3–*High-school-educated older adults*). Other classes, such as *High-school-educated employees* (C2) and *Non-compliant employees* (C9), showed relatively high acceptance rates, 67.77% and 65.47%, respectively. In the second round survey, the percentages of booster uptake showed a higher degree of variation, ranging from 42.2% (C4–*High-school-educated young adults*) to 74.85% (C6–*Educated older adults*). *Educated older adults* (C6) stood out with the highest percentage of booster uptake, while *High-school-educated young adults* (C4) showed the lowest percentage. The booster uptake rate was high in *High-school-educated older adults* (C3, 68.36%) and *University-educated employees* (C5, 62.45%). Conversely, *Unemployed* (C8) and *Non-compliant employees* (C9) showed lower uptake rates of 52.59% and 46.10%, respectively.

There were significant differences in the probabilities across latent classes. Compared with *University-educated older adults* (C4) with the highest rate of booster vaccine uptake in the second round, the probability of accepting COVID-19 vaccines was lowest in two classes, *Stay-at-home mothers* (C1) and *High-school-educated young adults* (C4). The probability of booster uptake was lowest among *High-school-educated young adults* (C4).

Specifically, the probability of accepting COVID-19 vaccines was 24 percentage points lower among *Stay-at-home mothers* (C1) [95% CI −0.36 to −0.13], 15 percentage points lower among *High-school-educated employees* (C2) [95% CI −0.26 to −0.04], 7 percentage points lower among *High-school-educated older adults* (C3) [95% CI −0.14 to −0.00], 24 percentage points lower among *High-school-educated young adults* (C4) [95% CI −0.36 to −0.11], 13 percentage points lower among *University-educated* *employees* (C5) [95% CI −0.22 to −0.03], 17 percentage points lower among *University-educated young adults* (C7) [95% CI −0.28 to −0.06], 21 percentage points lower among *Unemployed* (C8) [95% CI −0.34, −0.08], 20 percentage points lower among *Non-compliant employees* (C9) [95% CI −0.36 to −0.04] (Fig. 2A). The probability of booster uptake was 18 percentage points lower among *Stay-at-home mothers* (C1) [95% CI −0.29 to −0.07], 16 percentage points lower among *High-school-educated employees* (C2) [95% CI −0.29 to −0.04], 9 percentage points lower among *High-school-educated older adults* (C3) [95% CI -0.18 to -0.01], 29 percentage points lower among *High-school-educated young adults* (C4) [95% CI −0.41 to −0.17], 12 percentage points lower among *University-educated employees* (C5) [95% CI −0.24 to −0.01], 21 percentage points lower among *University-educated young adults* (C7) [95% CI −0.36 to −0.06], 20 percentage points lower among *Unemployed* (C8) [95% CI −0.33 to −0.07], 0.19 lower among *Non-compliant employees* (C9) [95% CI −0.33 to −0.06] (Fig. 2B).

There were 414 respondents who were misclassified: i.e., 328 respondents who were male, retirees, or students but classified as *stay-at-home mothers*, 41 students who were classified as *High-school-educated employees*, and 45 non-workers who were classified as *University-educated employees*. However, the results in Fig. 2 were robust against these misclassifications and replicable when excluding them (Supplementary Figure 3). When applying a different cutpoint of the vaccine acceptance outcome in round one (i.e., coding 1 if ‘definitely yes’ or ‘yes’), *University-educated older adults* still showed a higher probability of acceptance than most of the other classes (Supplementary Figure 4). However, the differences between this reference group and two classes–*University-educated employees* and *University-educated young adults*–became insignificant.

### The composition of vaccine-hesitant population by country

**Fig. 3:**
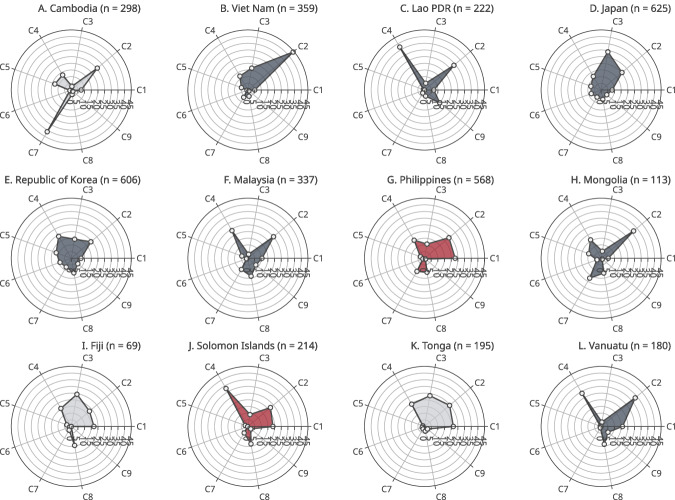
Latent class distributions among vaccine-hesitant respondents by country (June–August, 2021). Markers indicate weighted proportions of each latent class among respondents who answered that they did not want to receive a COVID-19 vaccine in round 1. Figures in parentheses are the number of respondents. C1–C9 indicate latent classes: C1–Stay-at-home mothers; C2–High-school-educated employees; C3–High-school-educated older adults; C4–High-school-educated young adults; C5–University-educated employees; C6–University-educated older adults; C7–University-educated young adults; C8–Unemployed; C9–Non-compliant employees. Colours indicate shared risk types: navy (plots **B**–**F**, **H**, **L**)–High-school-educated employees or High-school-educated younger adults; red (plot **G**, **J**)–Stay-at-home mothers; and grey (plots **A**, **I**, **K**)–no common risk types.

**Fig. 4:**
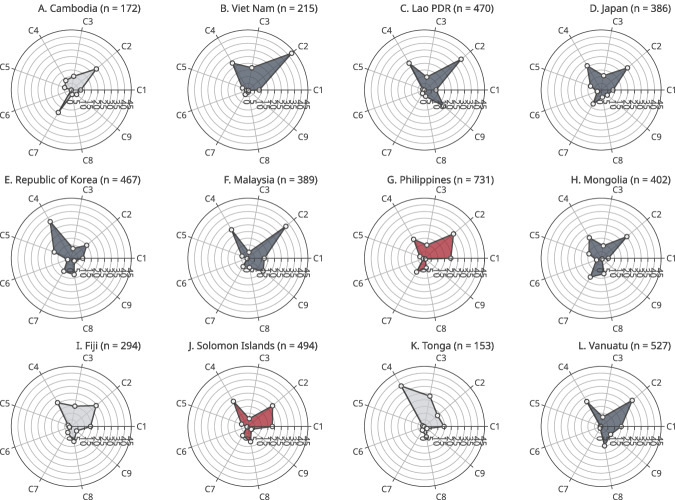
Latent class distributions among respondents without booster vaccine uptake by country (May–December, 2022). Markers indicate weighted proportions of each latent class among respondents who had not received COVID-19 booster vaccine dose in round 2. Figures in parentheses are the number of respondents. C1–C9 indicate latent classes: C1–Stay-at-home mothers; C2–High-school-educated employees; C3–High-school-educated older adults; C4–High-school-educated young adults; C5–University-educated employees; C6–University-educated older adults; C7–University-educated young adults; C8–Unemployed; C9–Non-compliant employees. Colours indicate shared risk types: navy (plots **B**–**F**, **H**, **L**)–High-school-educated employees or High-school-educated younger adults; red (plots **G**, **J**)–Stay-at-home mothers; and grey (plots **A**, **I**, **K**)–no common risk types.

Figures 3, 4 map out the varying profiles of vaccine-hesitant populations across countries and categorise countries into four groups based on shared risk factors in round two. In the figures, markers indicate the weighted proportion of each latent class among people who were unwilling to vaccinate for COVID-19 in round one and people who had not received a booster shot in round two. In general, *High-school-educated employees* (C2, 11 countries) and *High-school-educated young adults* (C4, 11 countries) took large portions in many countries.

The figures also show that each country had a unique profile of the vaccine-hesitant population. *High-school-educated employees* (C2) and *University-educated young adults* (C7) were the two largest latent classes in Cambodia (Fig. 4A). In Viet Nam, Lao PDR, Japan, the ROK, Malaysia, Mongolia and Vanuatu, the proportions of *High-school-educated employees* (C2) and *High-school-educated young adults* (C4) were relatively higher in the vaccine-hesitant population (Fig. 4B–F, H, L). *Stay-at-home mothers* (C1), *High-school-educated employees* (C2) and *High-school-educated young adults* (C4) took large proportions in the Philippines and Solomon Islands (Fig. 4G, J). These two latent classes took large portions in Fiji as well, however, there were also high proportions of *High-school-educated older adults* (C3) and *High-school-educated young adults* (C4) in this country (Fig. 4I). *University-educated employees* and the two classes of young adults took large proportions (Fig. 4E). Lastly, in Tonga, the proportions of *High-school-educated older adults* (C3) and *High-school-educated young adults* (C4) were high (Fig. 4K).

When compared to round two, the results from the round one survey examining vaccine acceptance largely shared similar patterns, however, there were also differences in several countries, indicating larger gaps between acceptance and actual booster uptake in some latent classes. For example, in Cambodia, the proportion of *University-educated young adults* (C7) was 36.08% among those who did not want to vaccinate in round one, however, it decreased to 19.45% among those without booster uptake in round two, indicating that people in this class were more likely to get a booster vaccine dose despite their initial vaccine hesitancy (Figs. 3A and 4A). In Viet Nam, the proportion of *High-school-educated young adults* (C4) was 11 percentage points higher in the second survey round (Figs. 3B and 4B). In Japan and the ROK, there was a higher proportion of *High-school-educated older adults* (C3) in round one, however, it decreased in round two by 18 percentage points in Japan and 7 percentage points in the ROK (Figs. 3D, E and 4D, E). In contrast, the proportion of *High-school-educated older adults* (C3) was higher by 9 percentage points in round one in Fiji (Figs. 3I and 4I). In Tonga, there were four latent classes with high proportion in round one: *Stay-at-home mothers* (C1) and *High-school-educated employees* (C2) took large proportions in round two, however, their proportions decreased in round 2 by 7 percentage points and 12 percentage points, respectively (Figs. 3K and 4K).

The unweighted results are presented in Supplementary Figs. 5 and 6. As the post-stratification weights accounted for discrepancies in educational attainment, there were differences in the latent class distribution between the main results and the unweighted results when it comes to six latent classes related to the level of education: *High-school-educated employees* (C2), *High-school-educated older adults* (C3), *High-school-educated young adults* (C4), *University-educated employees* (C5), *University-educated older adults* (C6) and *University-educated young adults* (C7).

## Discussion

This study examined the profile of vaccine-hesitant populations in 12 countries in the WPR by conducting LCA. Our analysis identified nine latent classes and demonstrated that each country presented the unique profile of the vaccine-hesitant population. There were also some latent classes that took large proportions across many countries, such as *High-school-educated employees* and *High-school-educated young adults*.

The country profiles based on smaller segments of latent classes can provide international and national policymakers with evidence for customising interventions to tackle vaccine hesitancy in the post-pandemic era. First, in most of the countries, where vaccine hesitancy was associated with those without tertiary-level education, immunisation campaigns can be tailored to target high-school-educated populations. For example, if the target audience is presumed to have lower levels of scientific literacy, programme designs could prioritise to incorporate simple language and visualisations, while providing more detailed and technical information for others. Such a messaging strategy would be more useful in countries where those without tertiary education took large proportions of not receiving booster uptake, such as Viet Nam, Lao PDR, Japan, ROK, Malaysia, Mongolia and Vanuatu.

Second, the Philippines and Solomon Islands shared a distinct profile of vaccine-hesitant people, where *stay-at-home mothers* represented a large portion of people without booster vaccine uptake. Prior studies found that low-risk perception of COVID-19 and concerns over vaccine safety and side effects are highly influential barriers for parents in vaccinating their children^49–51^. Furthermore, recent studies observed that social media information has a growing impact on vaccination choices of mothers and women of childbearing age^50,52^. Thus, measures to increase mothers’ understanding of the safety of vaccines may be beneficial in the Philippines and Solomon Islands. Considering that this class is likely to have closer connections with local or online communities than workplaces, vaccine-related messages or campaigns involving local health workers and community leaders may be a more effective means of combating vaccine hesitancy in these countries.

Third, other countries need to deal with a more complex profile with multiple subgroups. For example, in Cambodia, there were two distinct latent classes: *High-school-educated employees* and *University-educated young adults*. Two parallel strategies can be used in this country. First of all, the health authority can tailor its immunisation initiatives to disseminate information about vaccines through social media platforms for these young adults. In the meantime, it can also diversify the messages to target differing levels of scientific literacy for *High-school*-educated and educated populations.

An intervention with a narrowly defined target population provides several benefits. The intervention can deliver more effective and efficient solutions by incorporating tailored messages aimed at specific segments of the population, rather than dispersing efforts by attempting to reach a broader audience^53,54^. Moreover, an intervention targeting a smaller group can help reduce the risk of political backlash^55^. Therefore, the recommendations based on narrowly-defined target segments in this study can allow for a more strategic allocation of resources, enhancing both the precision and impact of the immunisation campaign while minimising unintended consequences or resistance from broader groups.

The suggested strategies should be in parallel with general efforts to investigate the country-specific causes of vaccine hesitancy and rebuild public confidence in vaccines and the health care system. The causes of vaccine hesitancy are highly contextual and vary by country, making an understanding of these factors crucial for interpreting the analysis results. For example, the 2017 Dengue vaccine scandal in the Philippines provides important context for understanding the profile of vaccine hesitancy in the country, where *stay-at-home mothers* were identified as the largest group among vaccine-hesitant people. It suggests that the profile of the vaccine-hesitant population—where *stay-at-home mothers* were identified as the largest share—may be linked to the increased parental hesitancy that has persisted since the scandal. Therefore, with additional efforts to understand the root causes and histories related to, the results from the subgroup analysis can be more interpretable and produce usable evidence for policymaking.

Lastly, more investigations and research are needed to develop more tailored interventions focusing on specific target groups. Only few empirical studies have been done focusing on developing countries and underrepresented populations, such as racial and ethnic minorities, in the WPR^31^. Future studies should focus on these populations that have not received sufficient attention in the scientific community.

This study has some limitations. First, our LCA approach estimated the maximum likelihood function at the regional level and then examined distributions at the national country level. Although we employed a fixed-effect approach including country categories as covariates, the number of identified latent classes might not maximise the likelihood function for each country and may vary by country. Despite this limitation, the international comparison in this paper still provides useful insight into how vaccine promotion strategies can be tailored based on varying profiles of vaccine-hesitant populations by generating comparable latent classes. Second, attempts to generalise the study’s findings require caution. Although we stratified the sampling to account for several demographic characteristics, this may not be sufficient to represent the vaccine-hesitant population, especially when it accounts for a small portion of the entire population. Considering a recent finding that countries in the WPR tend to show higher rates of COVID-19 vaccine confidence and uptake than some countries in Africa^56^, the profile of the vaccine-hesitant population can be more varied in other regions^56^. Moreover, the study data were collected in 2021 and 2022, when the threat from COVID-19 was still high in many countries, and there have been continuous changes in vaccine confidence across countries since the COVID-19 pandemic^57,58^. Therefore, attempts to use this study’s approach in the post-pandemic era should consider such varying contexts and changes over time. Lastly, we acknowledge that the study dataset was mixed with self-administered online surveys and computer-administered telephone surveys and could be exposed to potential biases, including selection bias and greater response rates to sensitive questions in online surveys^59^. This bias might affect the generalisability of the findings and the accuracy of responses toon sensitive items, such as vaccination status and willingness to vaccinate.

## Supplementary information

Supplementary material

## Data Availability

The raw data supporting the findings of this study are unavailable due to their sensitive nature, as requested by some of the participating countries.

## Code availability

Code supporting the findings of this study is available upon request.
