# Supplementary material for: Identifying vaccine-hesitant subgroups in the Western Pacific using latent class analysis
Source: NPJ Vaccines. 2025 Feb 12;10:29. doi: 10.1038/s41541-025-01067-3 (PMC11821871; doi:10.1038/s41541-025-01067-3)
Supplement: Supplementary file 1 — Supplementary material [file 41541_2025_1067_MOESM1_ESM.docx]

**Supplementary material for “Identifying Vaccine-hesitant Subgroups in the Western Pacific Using Latent Class Analysis”**

Yongjin Choi^1*^, Kathy Leung^2,3,4,5^, Joseph T. Wu^2,3,4,5^, Heidi J. Larson^1,6,7^, Leesa Lin^1,2,3^

^1^Department of Infectious Disease Epidemiology, London School of Hygiene and Tropical Medicine, London, United Kingdom

^2^Laboratory of Data Discovery for Health Limited (D24H), Hong Kong Science Park, Hong Kong SAR, China

^3^WHO Collaborating Centre for Infectious Disease Epidemiology and Control, School of Public Health, LKS Faculty of Medicine, The University of Hong Kong, Hong Kong SAR, China

^4^The University of Hong Kong – Shenzhen Hospital, Shenzhen, China

^5^The Hong Kong Jockey Club Global Health Institute, Hong Kong Special Administrative Region, P. R. China

^6^Centre for the Evaluation of Vaccination, Vaccine & Infectious Disease Institute, University of Antwerp, Antwerp, Belgium

^7^Department of Health Metrics Sciences, University of Washington, Seattle, WA, USA

*Corresponding author: Yongjin Choi, Department of Infectious Disease Epidemiology, London School of Hygiene and Tropical Medicine, Keppel St, London WC1E 7HT, United Kingdom; Email: [Yongjin.Choi@lshtm.ac.uk](mailto:Yongjin.Choi@lshtm.ac.uk)

# Supplementary Table 1. Data collection periods and methodologies

| **Country** | **Wave 1** | | **Wave 2** | | **Method** | **Language** |
| --- | --- | --- | --- | --- | --- | --- |
|  | **Sample** | **Dates (2021)** | **Sample** | **Dates (2022)** |  |  |
| Cambodia | 1,000 | June 14 – July 14 | 1,000 | May 17th – 26th | CATI | Khmer |
| Viet Nam | 1,044 | June 16th – July 14th | 1,003 | May 17th –26th | CATI | Vietnamese |
| Lao PDR | 1,000 | June 17 – July 11 | 1,000 | May 17th – 26th | CATI | Lao |
| Japan | 1,040 | July 5 - 8 | 1,066 | May 17th – 26th | Online | Japanese |
| Republic of Korea | 1,133 | July 5 – 14 | 1,155 | May 19th – June 1st | Online | Korean |
| Malaysia | 1,000 | June 14th – 27 | 1,000 | May 20th – June 20th | Online | Malay; English |
| Philippines | 1,000 | June 11 - July 4 | 1,000 | May 12th – 31st | CATI | Tagalog, Hiligaynon, Cebuano |
| Mongolia | 1,000 | June 22 – 30 | 1,000 | May 1st – 15th | CATI | Mongolian |
| Fiji | 521 | July 20 – August 7 | 508 | December 15th – 21st | CATI | Fijian; Fiji Hindi |
| Tonga | 517 | July 20 – August 10 | 206 | December 11th – 21st | CATI | Tongan |
| Solomon Islands | 527 | August 5 – 25 | 750 | December 13th – 22nd | CATI | English, Sols Pijin |
| Vanuatu | 532 | August 5 – 25 | 750 | December 11th – 21st | CATI | English, Vanuatu-Bislama |
| Total | 10,314 |  | 10,738 |  |  |  |

# Supplementary Table 2. Number of missing records before imputation by country

| **Variable** | **KHM** | **VNM** | **LAO** | **JPN** | **KOR** | **MYS** | **PHL** | **MNG** | **FJI** | **SLB** | **TON** | **VUT** |
| --- | --- | --- | --- | --- | --- | --- | --- | --- | --- | --- | --- | --- |
| **Round 1** |  |  |  |  |  |  |  |  |  |  |  |  |
| Willingness to vaccinate for COVID-19 | 0 | 0 | 0 | 0 | 0 | 0 | 0 | 0 | 0 | 0 | 0 | 0 |
| COVID-19 vaccination | 0 | 0 | 0 | 0 | 0 | 0 | 0 | 0 | 0 | 0 | 0 | 0 |
| COVID-19 risk perception | 0 | 0 | 0 | 0 | 0 | 0 | 0 | 0 | 0 | 0 | 0 | 0 |
| Mask wearing | 0 | 0 | 0 | 0 | 0 | 0 | 0 | 0 | 0 | 0 | 0 | 0 |
| Washing hands | 0 | 0 | 0 | 0 | 0 | 0 | 0 | 0 | 0 | 0 | 0 | 0 |
| Having guests | 0 | 0 | 0 | 0 | 0 | 0 | 0 | 0 | 0 | 0 | 0 | 0 |
| Sex | 0 | 0 | 0 | 18 | 76 | 0 | 0 | 0 | 0 | 0 | 0 | 0 |
| Age | 0 | 0 | 0 | 0 | 0 | 0 | 0 | 0 | 0 | 0 | 0 | 0 |
| Education | 0 | 0 | 0 | 70 | 100 | 10 | 2 | 0 | 2 | 2 | 3 | 5 |
| Employment status | 1 | 1 | 0 | 28 | 94 | 23 | 2 | 0 | 0 | 3 | 1 | 1 |
| **Round 2** |  |  |  |  |  |  |  |  |  |  |  |  |
| Willingness to vaccinate for COVID-19 | 0 | 0 | 0 | 0 | 0 | 0 | 0 | 0 | 0 | 0 | 0 | 0 |
| COVID-19 vaccination | 0 | 0 | 0 | 53 | 116 | 11 | 1 | 0 | 0 | 0 | 1 | 0 |
| COVID-19 risk perception | 0 | 0 | 0 | 0 | 0 | 0 | 0 | 0 | 9 | 13 | 8 | 38 |
| Mask wearing | 0 | 0 | 0 | 0 | 0 | 0 | 0 | 0 | 1 | 0 | 6 | 0 |
| Washing hands | 0 | 0 | 0 | 0 | 0 | 0 | 0 | 0 | 2 | 0 | 0 | 10 |
| Having guests | 0 | 0 | 0 | 0 | 0 | 0 | 0 | 0 | 1 | 1 | 0 | 7 |
| Sex | 0 | 0 | 0 | 57 | 152 | 0 | 0 | 0 | 0 | 1 | 0 | 11 |
| Age | 0 | 0 | 0 | 322 | 175 | 0 | 26 | 0 | 0 | 0 | 0 | 0 |
| Education | 9 | 0 | 0 | 66 | 162 | 19 | 1 | 3 | 7 | 0 | 0 | 0 |
| Employment status | 0 | 0 | 0 | 0 | 0 | 0 | 0 | 0 | 0 | 0 | 2 | 1 |

Note. KHM – Cambodia; VNM – Viet Nam; LAO – Lao PDR; JPN – Japan; Republic of Korea – KOR; MYS – Malaysia; PHL – The Philippines; MNG – Mongolia; FJI – Fiji; SLB – Solomon Islands; TON – Tonga; VUT – Vanuatu.

# Supplementary Table 3. Class labels before and after regrouping

| **Original class label** | **Class label after regrouping** | **Class ID** |
| --- | --- | --- |
| 1. Stay-at-home mothers | Stay-at-home mothers | 1 |
| 2. High-school-educated employees (35-54) | High-school-educated employees | 2 |
| 3. University-educated young adults (25-34) | University-educated young adults | 7 |
| 4. High-school-educated older adults | High-school-educated older adults | 3 |
| 5. University-educated older adults | University-educated older adults | 6 |
| 6. University-educated employees (18-24) | University-educated employees | 5 |
| 7. University-educated young adults (18-24) | University-educated young adults | 7 |
| 8. University-educated employees (35-44) | University-educated employees | 5 |
| 9. High-school-educated employees (25-34) | High-school-educated employees | 2 |
| 10. High-school-educated young adults (18-24) | High-school-educated young adults | 4 |
| 11. Unemployed | Unemployed | 8 |
| 12. Non-compliers | Non-compliers | 9 |

# Supplementary Table 4. Descriptive statistics (Unweighted)

| Variables | All respondents  (%) | Round 1  (%) | Round 2  (%) | Cramér's V |
| --- | --- | --- | --- | --- |
| Number of observations | 21,052 | 10,314 | 10,738 |  |
| Vaccine acceptance | - | 63.29 | - |  |
| Not vaccinated | - | - | 6.71 |  |
| Booster vaccination | - | - | 56.23 |  |
| Trust local health care providers | 83.60 | 82.71 | 84.45 | 0.0234 |
| Those who think that COVID-19 is exaggerated | 48.40 | 47.74 | 49.04 | 0.0130 |
| Health-protective behaviours |  |  |  |  |
| Mask wearing | 71.32 | 75.15 | 67.64 | 0.0830 |
| Washing hands | 80.04 | 83.12 | 77.08 | 0.0755 |
| Having guests | 39.47 | 17.68 | 60.41 | 0.4371 |
| Female | 49.56 | 49.54 | 49.57 | 0.0003 |
| Age |  |  |  | 0.0377 |
| 18-24 | 19.61 | 19.33 | 19.88 |  |
| 25-34 | 25.65 | 25.27 | 26.01 |  |
| 35-44 | 21.17 | 21.15 | 21.20 |  |
| 45-54 | 15.94 | 15.24 | 16.60 |  |
| 55+ | 17.63 | 19.01 | 16.31 |  |
| Education |  |  |  | 0.0217 |
| Tertiary or lower | 64.83 | 65.40 | 64.28 |  |
| Tertiary | 31.05 | 30.19 | 31.88 |  |
| Master+ | 4.12 | 4.41 | 3.85 |  |
| Employment status |  |  |  | 0.0353 |
| Employed | 59.98 | 59.76 | 60.18 |  |
| Students | 7.51 | 8.18 | 6.87 |  |
| Retirees | 5.90 | 6.36 | 5.46 |  |
| Stay-at-home parents | 13.91 | 13.32 | 14.47 |  |
| Unemployed or others | 12.70 | 12.37 | 13.02 |  |

# Supplementary Table 5. Sample characteristics by country – Round 1

| **Variable** | **KHM**  **(%)** | **VNM**  **(%)** | **LAO**  **(%)** | **JPN**  **(%)** | **KOR**  **(%)** | **MYS**  **(%)** | **PHL**  **(%)** | **MNG**  **(%)** | **FJI**  **(%)** | **SLB**  **(%)** | **TON**  **(%)** | **VUT**  **(%)** |
| --- | --- | --- | --- | --- | --- | --- | --- | --- | --- | --- | --- | --- |
| Number of observations | 1,000 | 1,044 | 1,000 | 1040 | 1,133 | 1,000 | 1,000 | 1,000 | 521 | 527 | 517 | 532 |
| Vaccine acceptance | 70.20 | 65.61 | 77.80 | 39.90 | 46.51 | 66.30 | 43.20 | 88.70 | 86.76 | 59.39 | 62.28 | 66.17 |
| Trust local health care providers | 90.70 | 95.11 | 64.70 | 78.56 | 72.20 | 80.20 | 81.90 | 92.20 | 84.26 | 81.02 | 95.94 | 83.46 |
| Those who think that COVID-19 is exaggerated | 27.30 | 23.18 | 64.70 | 40.29 | 33.10 | 57.90 | 53.20 | 48.50 | 62.76 | 58.06 | 98.45 | 43.23 |
| **Health-protective behaviours** |  |  |  |  |  |  |  |  |  |  |  |  |
| Mask wearing | 88.70 | 97.70 | 52.30 | 85.87 | 81.11 | 78.70 | 78.80 | 96.40 | 86.95 | 27.89 | 45.45 | 25.38 |
| Washing hands | 88.70 | 96.55 | 50.70 | 79.52 | 80.41 | 81.40 | 91.20 | 92.30 | 95.01 | 79.13 | 90.52 | 75.94 |
| Having guests | 19.30 | 2.30 | 22.10 | 12.79 | 12.36 | 9.70 | 26.60 | 4.70 | 11.52 | 43.64 | 46.81 | 31.95 |
| Female | 50.90 | 49.52 | 49.60 | 51.35 | 48.01 | 49.80 | 50.00 | 51.30 | 49.71 | 42.88 | 50.10 | 47.93 |
| **Age** |  |  |  |  |  |  |  |  |  |  |  |  |
| 18-24 | 20.00 | 12.07 | 26.50 | 10.38 | 16.24 | 24.20 | 22.70 | 15.60 | 19.19 | 27.13 | 21.08 | 25.19 |
| 25-34 | 31.90 | 25.00 | 29.10 | 12.31 | 15.53 | 35.10 | 26.00 | 27.30 | 24.76 | 27.70 | 23.79 | 28.01 |
| 35-44 | 23.40 | 24.90 | 19.70 | 15.10 | 15.98 | 26.40 | 23.10 | 22.40 | 20.92 | 20.30 | 20.89 | 20.49 |
| 45-54 | 16.60 | 17.53 | 13.90 | 16.73 | 17.74 | 8.50 | 14.60 | 17.10 | 14.78 | 15.56 | 8.90 | 19.17 |
| 55+ | 8.10 | 20.50 | 10.80 | 45.48 | 34.51 | 5.80 | 13.60 | 17.60 | 20.35 | 9.30 | 25.34 | 7.14 |
| **Education** |  |  |  |  |  |  |  |  |  |  |  |  |
| Tertiary or lower | 86.00 | 75.10 | 92.00 | 45.58 | 39.01 | 41.50 | 74.40 | 56.10 | 58.54 | 71.92 | 82.98 | 81.20 |
| Tertiary | 13.80 | 23.75 | 6.80 | 43.37 | 44.84 | 53.40 | 24.00 | 41.20 | 38.39 | 26.76 | 16.63 | 16.54 |
| Master+ | 0.20 | 1.15 | 1.20 | 11.06 | 16.15 | 5.10 | 1.60 | 2.70 | 3.07 | 1.33 | 0.39 | 2.26 |
| **Employment status** |  |  |  |  |  |  |  |  |  |  |  |  |
| Employed | 72.40 | 72.61 | 68.80 | 56.25 | 59.31 | 70.50 | 44.00 | 63.40 | 41.46 | 41.75 | 44.68 | 54.70 |
| Students | 4.50 | 5.84 | 12.30 | 3.94 | 6.44 | 7.20 | 10.30 | 7.20 | 10.36 | 17.08 | 8.32 | 12.59 |
| Retirees | 0.40 | 6.42 | 1.40 | 10.29 | 9.00 | 1.40 | 1.60 | 13.60 | 12.09 | 4.74 | 17.79 | 3.01 |
| Stay-at-home parents | 10.10 | 9.39 | 10.30 | 18.27 | 12.18 | 7.60 | 24.90 | 5.50 | 17.66 | 17.08 | 20.89 | 13.91 |
| Unemployed or others | 12.60 | 5.75 | 7.20 | 11.25 | 13.06 | 13.30 | 19.20 | 10.30 | 18.43 | 19.35 | 8.32 | 15.79 |

Note. KHM – Cambodia; VNM – Viet Nam; LAO – Lao PDR; JPN – Japan; Republic of Korea – KOR; MYS – Malaysia; PHL – The Philippines; MNG – Mongolia; FJI – Fiji; SLB – Solomon Islands; TON – Tonga; VUT – Vanuatu.

# Supplementary Table 6. Sample characteristics by country – Round 2

| **Variable** | **KHM**  **(%)** | **VNM**  **(%)** | **LAO**  **(%)** | **JPN**  **(%)** | **KOR**  **(%)** | **MYS**  **(%)** | **PHL**  **(%)** | **MNG**  **(%)** | **FJI**  **(%)** | **SLB**  **(%)** | **TON**  **(%)** | **VUT**  **(%)** |
| --- | --- | --- | --- | --- | --- | --- | --- | --- | --- | --- | --- | --- |
| Number of observations | 1,000 | 1,003 | 1,000 | 1,066 | 1,155 | 1,000 | 1,000 | 1,000 | 508 | 750 | 506 | 750 |
| COVID-19 vaccination – 1 dose or more | 98.30 | 99.90 | 98.50 | 87.71 | 87.79 | 97.40 | 92.20 | 96.30 | 94.49 | 83.47 | 97.83 | 85.20 |
| COVID-19 vaccination – 3 doses or more | 82.80 | 78.56 | 53.00 | 63.79 | 59.57 | 61.10 | 26.90 | 59.80 | 42.13 | 34.13 | 69.76 | 29.73 |
| Trust local health care providers | 97.70 | 98.40 | 91.10 | 74.02 | 66.41 | 79.10 | 90.50 | 91.70 | 86.61 | 68.00 | 93.48 | 80.13 |
| Those who think that COVID-19 is exaggerated | 11.20 | 26.32 | 67.80 | 50.66 | 32.12 | 55.30 | 50.70 | 52.00 | 70.67 | 56.00 | 72.92 | 76.40 |
| **Health-protective behaviours** |  |  |  |  |  |  |  |  |  |  |  |  |
| Mask wearing | 68.50 | 96.81 | 53.10 | 82.74 | 72.21 | 71.90 | 80.40 | 77.80 | 55.51 | 37.60 | 66.01 | 21.47 |
| Washing hands | 69.60 | 96.11 | 52.40 | 76.92 | 71.43 | 72.60 | 90.20 | 94.70 | 79.53 | 76.00 | 89.33 | 59.60 |
| Having guests | 41.50 | 77.17 | 46.20 | 70.73 | 68.57 | 67.60 | 47.60 | 79.90 | 53.35 | 59.20 | 71.15 | 35.20 |
| Female | 51.00 | 50.75 | 45.90 | 51.03 | 48.14 | 49.60 | 49.90 | 51.30 | 49.02 | 49.07 | 51.19 | 48.13 |
| **Age** |  |  |  |  |  |  |  |  |  |  |  |  |
| 18-24 | 18.00 | 12.66 | 20.80 | 16.04 | 22.08 | 24.90 | 21.20 | 15.60 | 21.06 | 23.33 | 21.34 | 24.93 |
| 25-34 | 27.00 | 25.22 | 30.60 | 18.01 | 18.61 | 32.50 | 27.80 | 27.30 | 25.00 | 29.73 | 24.11 | 27.87 |
| 35-44 | 20.30 | 22.03 | 21.10 | 19.23 | 17.92 | 27.10 | 21.90 | 22.40 | 20.28 | 21.07 | 19.76 | 20.53 |
| 45-54 | 14.80 | 18.15 | 16.40 | 21.20 | 18.96 | 11.30 | 16.80 | 17.10 | 15.55 | 17.60 | 10.47 | 17.07 |
| 55+ | 19.90 | 21.93 | 11.10 | 25.52 | 22.42 | 4.20 | 12.30 | 17.60 | 18.11 | 8.27 | 24.31 | 9.60 |
| **Education** |  |  |  |  |  |  |  |  |  |  |  |  |
| Tertiary or lower | 85.00 | 77.87 | 63.60 | 51.59 | 38.10 | 38.40 | 74.00 | 59.40 | 65.35 | 74.53 | 77.67 | 85.73 |
| Tertiary | 13.90 | 21.64 | 35.50 | 42.40 | 46.32 | 54.10 | 25.60 | 37.70 | 32.48 | 24.67 | 19.57 | 13.60 |
| Master+ | 1.10 | 0.50 | 0.90 | 6.00 | 15.58 | 7.50 | 0.40 | 2.90 | 2.17 | 0.80 | 2.77 | 0.67 |
| **Employment status** |  |  |  |  |  |  |  |  |  |  |  |  |
| Employed | 72.20 | 74.28 | 75.90 | 59.38 | 54.72 | 78.70 | 48.40 | 59.90 | 50.59 | 44.80 | 32.21 | 46.00 |
| Students | 4.10 | 5.98 | 5.40 | 3.75 | 5.71 | 4.40 | 10.10 | 8.20 | 8.66 | 11.87 | 8.50 | 9.87 |
| Retirees | 1.40 | 6.88 | 2.20 | 10.51 | 6.49 | 0.70 | 0.90 | 13.40 | 8.46 | 4.00 | 7.11 | 4.67 |
| Stay-at-home parents | 19.20 | 11.67 | 4.70 | 13.51 | 12.03 | 5.30 | 24.90 | 5.20 | 17.32 | 20.93 | 39.33 | 15.60 |
| Unemployed or others | 3.10 | 1.20 | 11.80 | 12.85 | 21.04 | 10.90 | 15.70 | 13.30 | 14.96 | 18.40 | 12.85 | 23.87 |

Note. KHM – Cambodia; VNM – Viet Nam; LAO – Lao PDR; JPN – Japan; Republic of Korea – KOR; MYS – Malaysia; PHL – The Philippines; MNG – Mongolia; FJI – Fiji; SLB – Solomon Islands; TON – Tonga; VUT – Vanuatu.

# Supplementary Table 7. Comparison of COVID-19 vaccination rate

| **Country** | **Study sample (weighted)** | | **Benchmark data1** | |
| --- | --- | --- | --- | --- |
|  | **%** | **Dates (2022)** | **%** | **Date (2022)** |
| Cambodia | 98.91 | May 17th – 26th | 89.59 | May 20th |
| Viet Nam | 99.89 | May 17th –26th | 85.78 | May 26th |
| Lao PDR | 98.30 | May 17th – 26th | 77.12 | May 26th |
| Japan | 87.68 | May 17th – 26th | 83.56 | May 26th |
| Republic of Korea | 85.35 | May 19th – June 1st | 86.28 | June 1st |
| Malaysia | 97.00 | May 20th – June 20th | 82.69 | June 20th |
| Philippines | 91.78 | May 12th – 31st | 64.59 | June 5th |
| Mongolia | 96.34 | May 1st – 15th | 66.88 | May 11th |
| Fiji | 93.49 | December 15th – 21st | 76.53 | December 19th |
| Tonga | 98.12 | December 11th – 21st | 81.66 | November 28th |
| Solomon Islands | 82.00 | December 13th – 22nd | - | Data not available within one month |
| Vanuatu | 84.58 | December 11th – 21st | - | Data not available within one month |

Note. The benchmark data used for comparison is sourced from the ‘Coronavirus (COVID-19) Vaccinations’ section on the Our World in Data website, accessible at: ourworldindata.org/covid-vaccinations.

# Supplementary Figure 1. Scree plots of latent class analysis


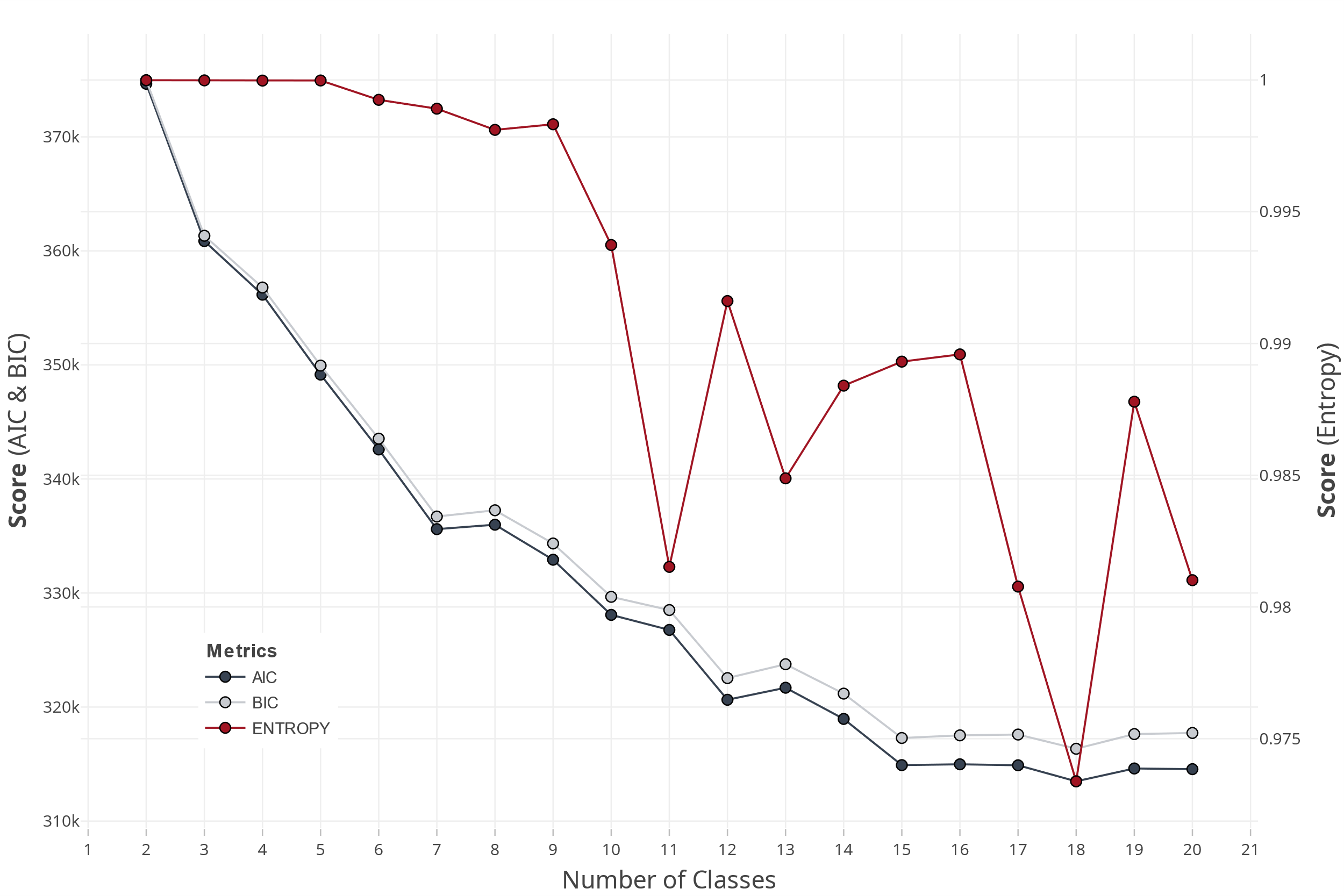


Note. Markers indicate the scores of fit indices. The Y-axis on the left represents the AIC and BIC scores, while the secondary Y-axis on the right represents the entropy score.

# Supplementary Figure 2. Class distribution (n = 21,052)


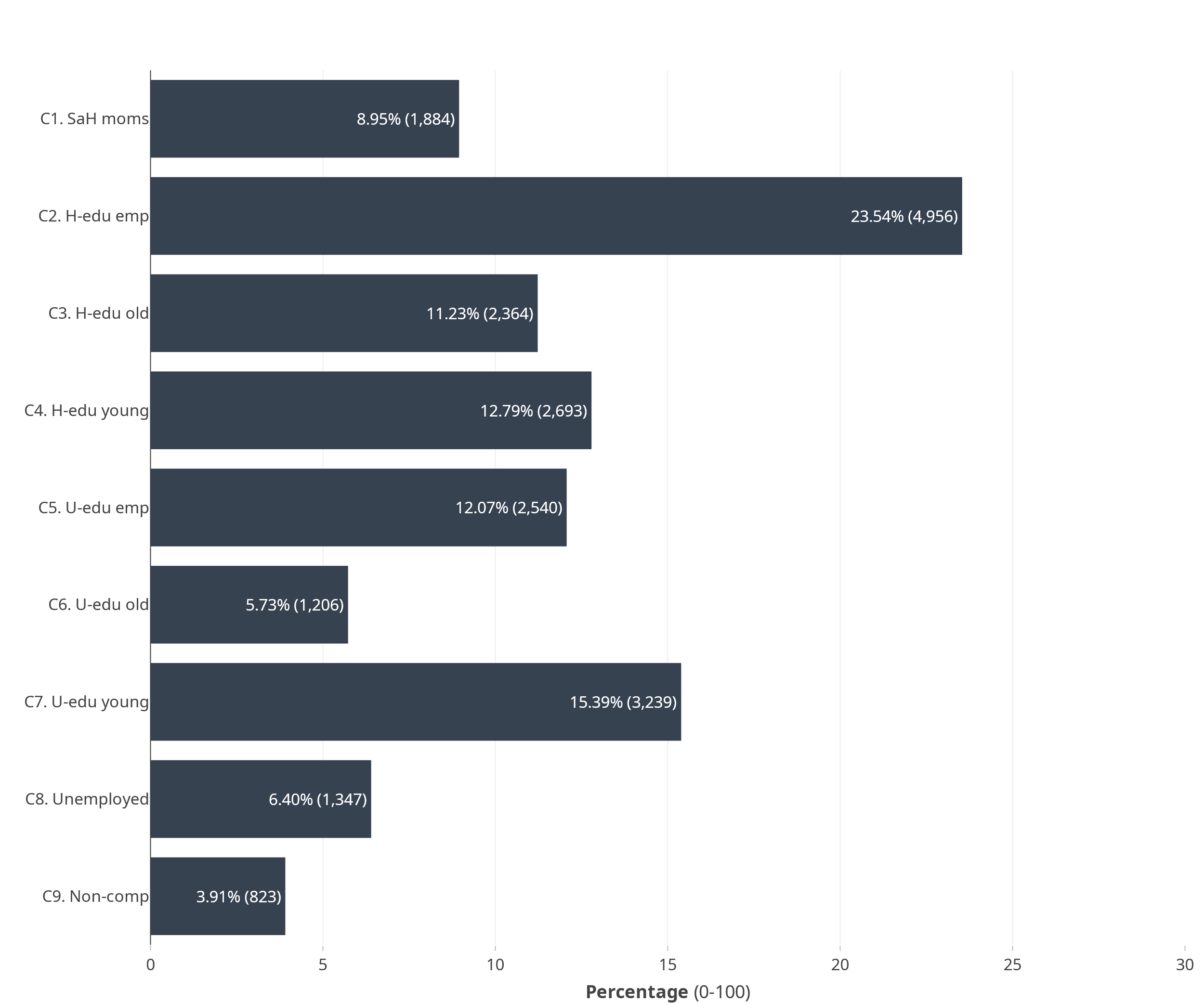


Note. C1-C9 indicate latent classes: C1-C9 indicate latent classes: C1 – Stay-at-home mothers; C2 – High-school-educated employees; C3 – High-school-educated older adults; C4 – High-school-educated young adults; C5 – University-educated employees; C6 – University-educated older adults; C7 – University-educated young adults; C8 – Unemployed; C9 – Non-compliant employees.

# Supplementary Figure 3. Comparison of COVID-19 vaccine acceptance and booster uptake by latent class after excluding misclassified observations


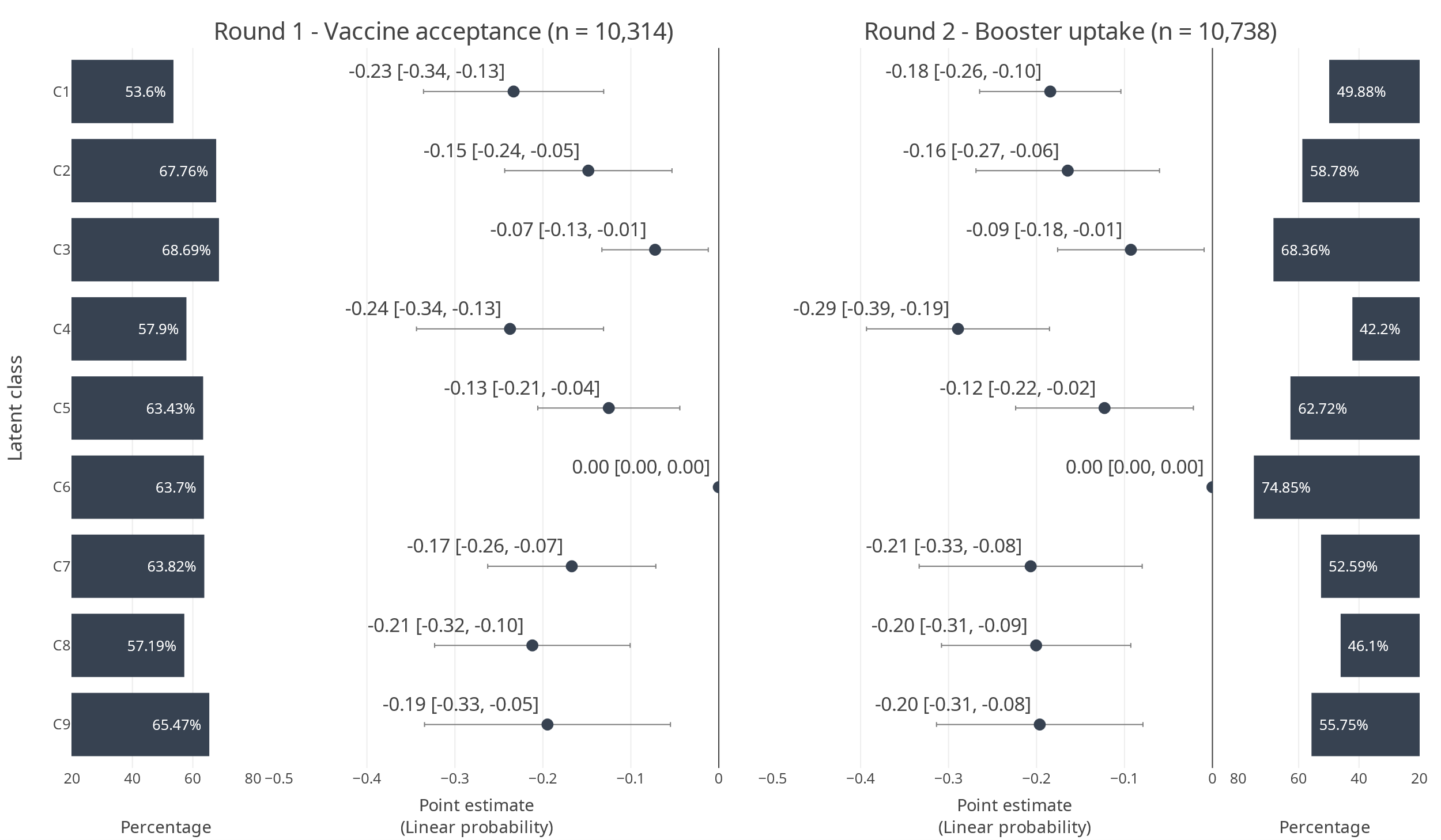


Note. Markers indicate OLS coefficients. C1-C9 indicate latent classes: C1-C9 indicate latent classes: C1 – Stay-at-home mothers; C2 – High-school-educated employees; C3 – High-school-educated older adults; C4 – High-school-educated young adults; C5 – University-educated employees; C6 – University-educated older adults; C7 – University-educated young adults; C8 – Unemployed; C9 – Non-compliant employees. Horizontal spikes around the markers indicate 95% CIs. Unobserved country-level variations were fixed. Standard errors were clustered at the country level.

# Supplementary Figure 4. COVID-19 vaccine acceptance by latent class with a different cutpoint (n = 10,314)


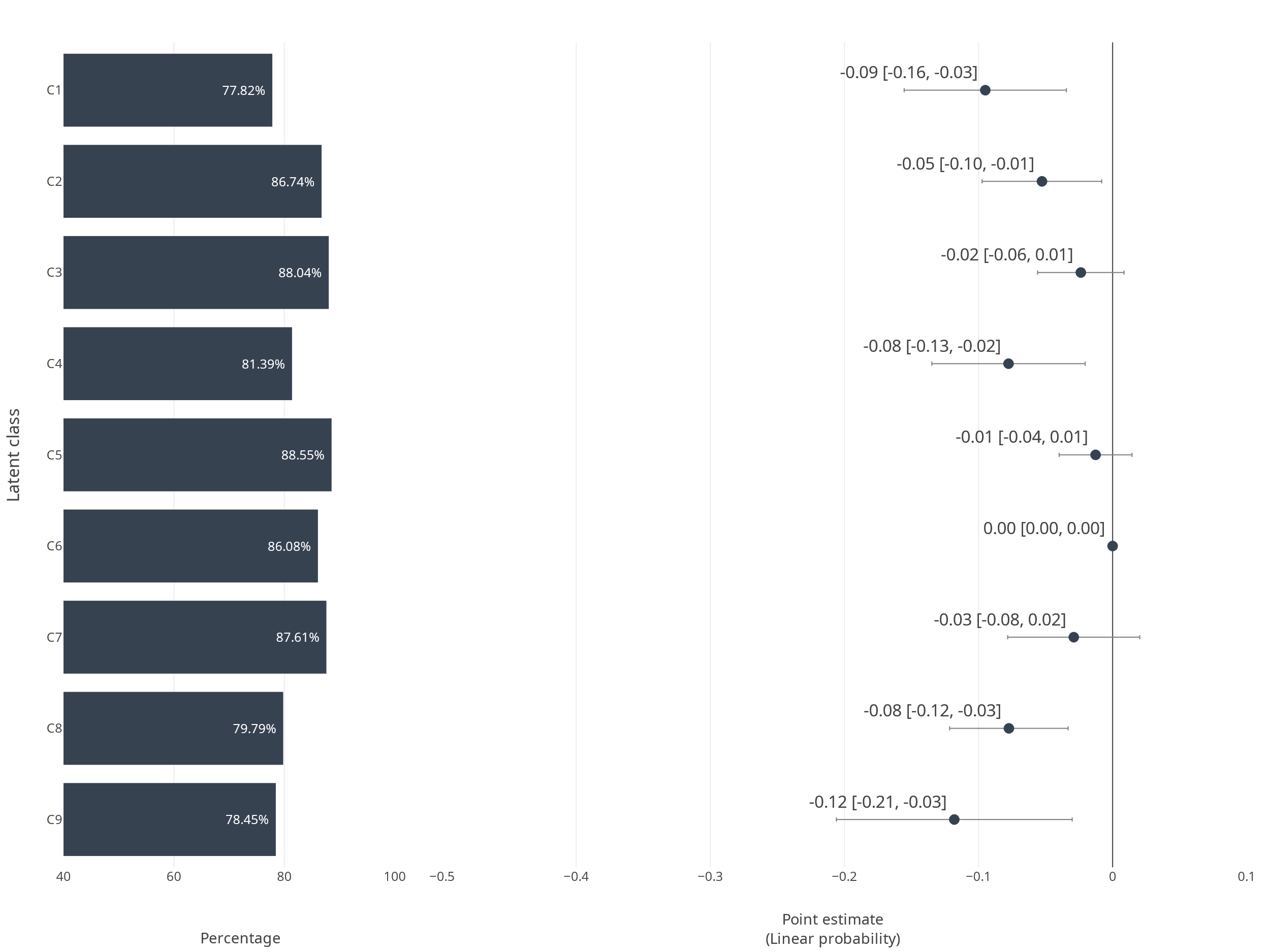


Note. Markers indicate OLS coefficients. C1-C9 indicate latent classes: C1-C9 indicate latent classes: C1 – Stay-at-home mothers; C2 – High-school-educated employees; C3 – High-school-educated older adults; C4 – High-school-educated young adults; C5 – University-educated employees; C6 – University-educated older adults; C7 – University-educated young adults; C8 – Unemployed; C9 – Non-compliant employees. Horizontal spikes around the markers indicate 95% CIs. Unobserved country-level variations were fixed. Standard errors were clustered at the country level. The outcome variable is the acceptance of COVID-19 vaccines, which was coded as 1 if respondents answered “definitely yes” or “yes.” This result is based on a complete case analysis.

# Supplementary Figure 5. Latent class distributions among vaccine-hesitant respondents by country (unweighted)


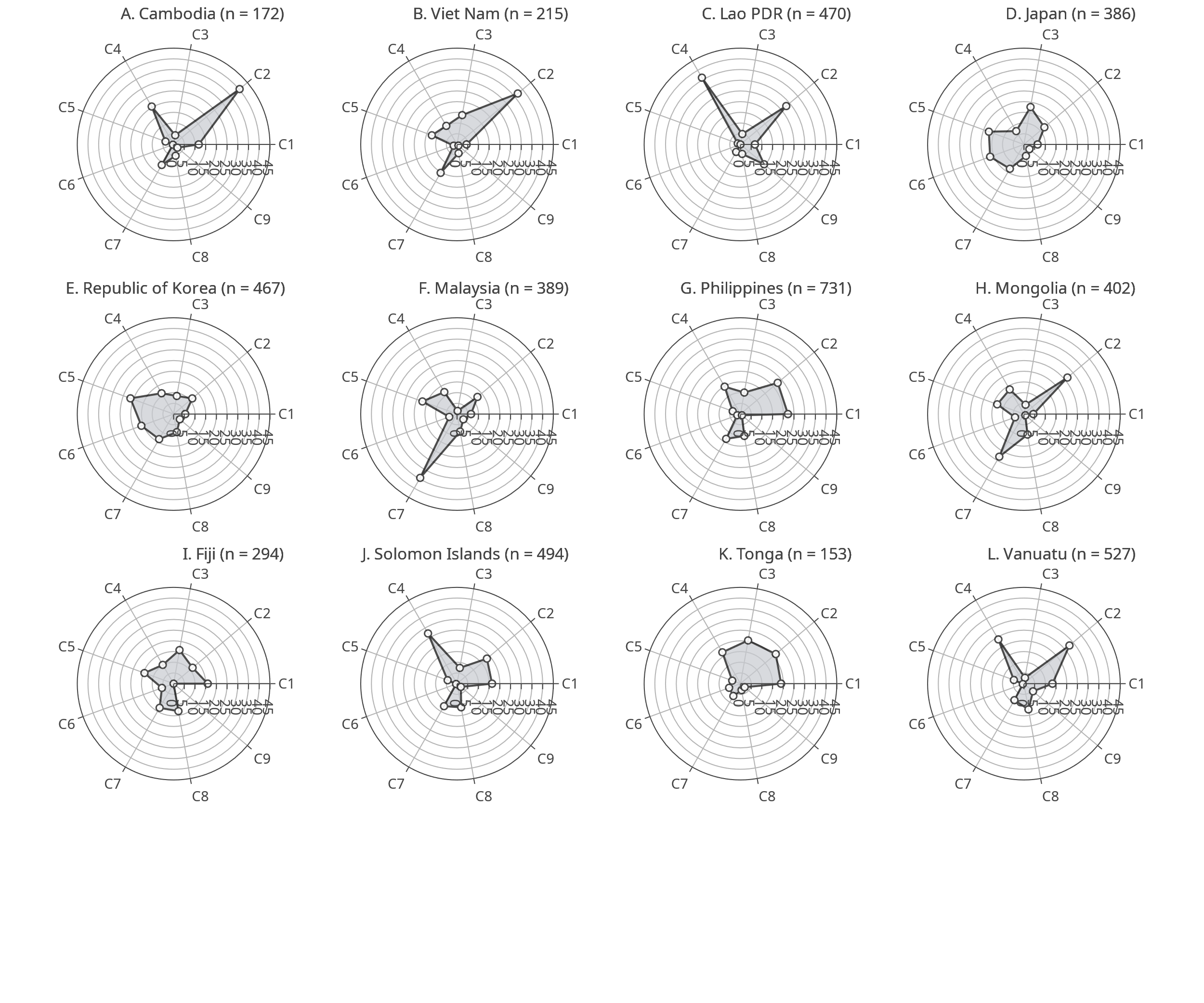


Note. Markers indicate percentages. Figures in parentheses are the number of respondents who were not willing to vaccinate for COVID-19. C1-C9 indicate latent classes: C1-C9 indicate latent classes: C1 – Stay-at-home mothers; C2 – High-school-educated employees; C3 – High-school-educated older adults; C4 – High-school-educated young adults; C5 – University-educated employees; C6 – University-educated older adults; C7 – University-educated young adults; C8 – Unemployed; C9 – Non-compliant employees.

# Supplementary Figure 6. COVID-19 vaccine acceptance by latent class with a different cutpoint (n = 9,878)


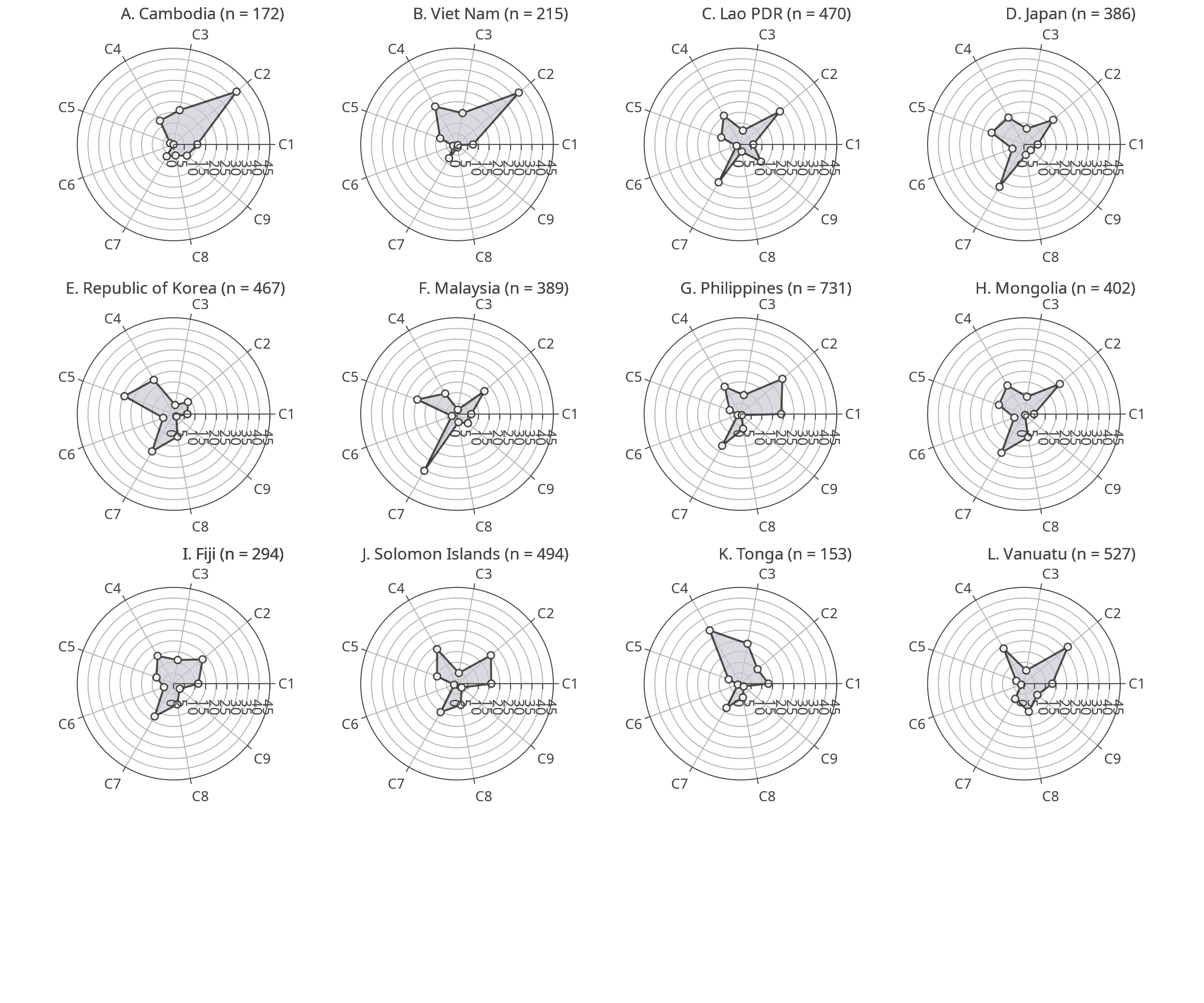


Note. Markers indicate percentages. Figures in parentheses are the number of respondents who were not willing to vaccinate for COVID-19. C1-C9 indicate latent classes: C1-C9 indicate latent classes: C1 – Stay-at-home mothers; C2 – High-school-educated employees; C3 – High-school-educated older adults; C4 – High-school-educated young adults; C5 – University-employees; C6 – University-educated older adults; C7 – University-educated young adults; C8 – Unemployed; C9 – Non-compliant employees.
